# Infectious Spondylodiscitis of Bacterial Causes in Adults: Epidemiology, Pathophysiology, Diagnostic and Treatment Challenges

**DOI:** 10.3390/microorganisms14051110

**Published:** 2026-05-13

**Authors:** Bogdan Sendrea, Argyrios Periferakis, Aristodemos-Theodoros Periferakis, Ioannis Xefteris, Lamprini Troumpata, Konstantinos Periferakis, Andreea-Elena Scheau, Emi Marinela Preda, Dana-Georgiana Nedelea, Diana-Elena Vulpe, Rares-Mircea Birlutiu, Cristian Scheau, Romica Cergan

**Affiliations:** 1 Faculty of Medicine, The “Carol Davila” University of Medicine and Pharmacy, 050474 Bucharest, Romania; 2 “Foisor” Clinical Hospital of Orthopaedics, Traumatology and Osteoarticular TB, 021382 Bucharest, Romania; 3 Akadimia of Ancient Greek and Traditional Chinese Medicine, 16675 Athens, Greece; 4 Elkyda, Research & Education Centre of Charismatheia, 17675 Athens, Greece; 5 Pan-Hellenic Organization of Educational Programs (P.O.E.P.), 17236 Athens, Greece

**Keywords:** infectious spondylodiscitis, pyogenic spondylodiscitis, granulomatous spondylodiscitis, Pott’s disease, diagnostic work-up, medical imaging, pathophysiology, therapeutic approach, patient management

## Abstract

Spinal infections in general, and infectious spondylodiscitis in particular, are increasingly diagnosed in the Western world, in recent decades. This rise in incidence is associated with an ageing population and with an increased availability of accurate diagnostic modalities. Even so, due to the non-specific nature of clinical manifestations, and of the implicated blood and serum markers, there is a risk of underdiagnosis or misdiagnosis of the disease in its initial stages. Ionizing radiation methods, such as plain radiography (X-ray) and computed tomography (CT), are also not reliable in the early stages of the diseases, and the golden standard of imagistic diagnosis, magnetic resonance imaging (MRI), is not always available or requested. Still, MRI remains the most reliable method in most cases where there is a need for differential diagnosis with other pathologies, namely Andersson lesions, destructive spondyloarthropathy, erosive osteochondritis, micro-crystalline spondylitis, Modic 1 lesion, Charcot spinal arthropathy, osteoporotic fractures, SAPHO syndrome with spinal involvement, and Schmorl’s nodes. Infectious spondylodiscitis is caused by bacteria, and, less frequently, by fungi. Rare cases of parasitic causes have also been reported in the literature. Infectious spondylodiscitis of bacterial causes may be pyogenic, more frequently caused by *Staphylococcus* spp. or *Streptococcus* spp., or granulomatous, usually caused by *Mycobacterium tuberculosis* complex (MTBC) or from classical brucellosis. In all these cases, therapy may be conservative, with antibiotics, or surgical, when the former fails or in patients with significant spinal instability or other neurological manifestations. There are various surgical approaches, each with its own drawbacks, and usually used according to the preference of the attending physician. Even in cases of surgical treatment, antibiotic administration is prolonged, and it is important for a proper scheme to be selected based on antimicrobial susceptibility testing. However, given that in many cases, the causative agent cannot be identified, empirical treatment must be initiated. Finally, newer approaches, including the incorporation of antimicrobial substances, may offer better solutions for improving treatment and rehabilitation outcomes.

## 1. Introduction

Pyogenic spinal infections appear to be experiencing an upward trend, especially in Europe; this is believed to be attributable to several factors [1,2,3]. This increase, coupled with complexities and uncertainties in diagnosis and treatment lead to rising healthcare costs [4,5]. The spine comprises one of relatively frequent sites of infection, accounting for about 2–7% of all osteoarticular infections [6,7,8]. The main clinical presentation of spinal infections is infectious spondylodiscitis, a term comprising different clinical entities; the pyogenic form is the more common one, comprising up to 80% of cases, depending on the region [9]. While having come to the foreground relatively recently, due to an increase in incidence, even in modern healthcare systems, it is an ancient disease, with evidence having been found in human remains from the 7–8th century BC [10].

Infectious spondylodiscitis, also known as vertebral osteomyelitis, is defined as an infection of the vertebral bodies and intervertebral discs, caused by pyogenic bacteria [11]. It is a severe condition, oftentimes leading to hospitalization [12,13,14], and causing significant morbidity and mortality [9]. The complexities in treatment and the probability of long-term sequalae indicate the need for prompt diagnosis and rapid treatment initiation; however, both diagnosis and treatment present challenges of their own.

While infectious spondylodiscitis has been addressed in previous reviews, this paper aims to provide an updated clinically oriented synthesis, integrating recent epidemiological trends, etiologies, differential diagnosis, limitations in molecular and microbiological diagnosis, as well as challenges in treatment. The following sections provide a structured reference for clinicians facing difficulties and uncertainty in diagnosis, managing culture-negative disease, or facing complex therapeutic decisions.

### 1.1. Study Design

This study was conducted as a structured narrative review with systematic literature identification, designed to synthesize current evidence on infectious spondylodiscitis. The methodology followed the principles of the PRISMA (Preferred Reporting Items for Systematic Reviews and Meta-Analyses) 2020 guidelines to enhance transparency and reproducibility. In this review, we will present the current epidemiological data on infectious spondylodiscitis, explain the pathophysiology and clinical manifestations, discuss the aetiologic factors associated with spondylodiscitis, and present the challenges associated with treatment and diagnosis. In this way, we aim to provide a comprehensive overview of this clinical entity to serve as a guide for future research, both in terms of diagnosis and treatment. We have chosen to focus only on infectious spondylodiscitis in adults, as there exists more data in the relevant medical literature on its epidemiology, diagnosis, and treatment, and also because spinal infections in children are complex clinical entities [15], presenting with a triphasic age distribution [16], and, as such, merit a more specific and comprehensive focus.

### 1.2. Literature Search Strategy

A comprehensive literature search was performed in PubMed, Embase, and Scopus to identify relevant studies published between January 2000 and December 2025. The search strategy included combinations of the following keywords: “spondylodiscitis”, “vertebral osteomyelitis”, “discitis”, and “spinal infection”. To ensure completeness, additional studies were identified through manual screening of the reference lists of eligible articles. A limited number of older studies were retained due to their recognized seminal or historical importance.

### 1.3. Eligibility Criteria

Studies were included if they met the following criteria: addressed bacterial infectious spondylodiscitis in adult populations; reported data relevant to epidemiology, pathophysiology, diagnosis, or treatment; and were original studies, reviews, or clinically relevant reports.

Exclusion criteria were: fungal or parasitic infections unless specifically relevant to the discussion, pediatric populations or mixed cohorts without separate adult analysis, insufficient relevance to the objectives of the review, and unavailable or incomplete full-text data. Tuberculosis and brucellar spondylodiscitis were retained when addressed as bacterial/granulomatous forms of infectious spondylodiscitis.

### 1.4. Study Selection

All identified records were exported into a reference management software, and duplicates were removed using a combination of automated detection and manual verification. A total of 1973 records were identified, including 1927 from database searching (PubMed, *n* = 512; Embase, *n* = 684; Scopus, *n* = 731) and 46 from reference list screening. After the removal of 624 duplicates, 1349 records remained for title and abstract screening.

Screening and eligibility assessments were performed independently by two reviewers. Of these, 841 records were excluded as clearly outside the scope of the review. A total of 508 full-text articles were assessed for eligibility, of which 206 were excluded for predefined reasons, including non-bacterial etiology (*n* = 58), pediatric or mixed populations without separate adult analysis (*n* = 32), insufficient relevance (*n* = 77), and unavailable full-text data (*n* = 39).

**Figure 1:**
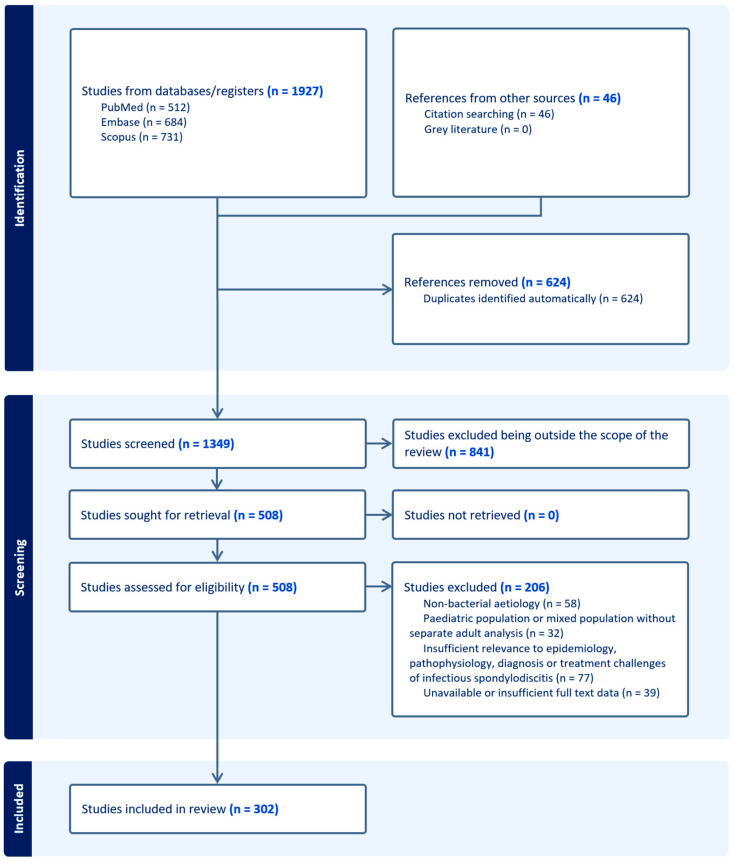
PRISMA flow diagram illustrating the study selection process.

Ultimately, 302 studies were included in the qualitative synthesis. All identified records were exported into a reference management software, and duplicates were removed through a combination of automated and manual processes. Title and abstract screening, followed by full-text eligibility assessment, was independently performed by two reviewers. Discrepancies were resolved through discussion and consensus. The study selection process is summarized in Figure 1 (PRISMA flow diagram).

### 1.5. Data Extraction

Relevant data were extracted using a standardized approach. Data extraction was performed by one reviewer and verified by a second reviewer to ensure accuracy. Risk of bias assessment was performed by one reviewer and independently verified by a second reviewer, with discrepancies resolved through discussion and consensus. Due to the heterogeneity in the design of the included literature studies, risk of bias was assessed qualitatively rather that using scoring instruments, considering the study design and clarity of methodology and diagnostic criteria, method of microbiological confirmation, clinical and imaging data completeness, sample size and selection bias, as well as consistency and clarity of reported treatment and outcomes. Due to the narrative nature of this review and the large number of included studies, the risk of bias assessment was not used as an exclusion criterion, but as an instrument to contextualize the evidence, keeping with the narrative purpose of the review. Common limitations included retrospective study designs, relatively small sample sizes, heterogeneity in diagnostic criteria and treatment protocols.

## 2. Epidemiology of Infectious Spondylodiscitis: Incidence, Mortality, and Risk Factors

There is an apparent increase in cases of infectious spondylodiscitis, at least in Europe. For example, in France, between 2010 and 2019, its incidence rose from 6.1/10^5^ to 11.3/10^5^ [2]. A similarly high incidence was documented in England and Germany in recent years, with most cases being in elderly patients; the rise in admissions for this particular pathology was 44% in England [17] and 104% in Germany [18]. In the previous decade, between 1995 and 2008, the incidence in Denmark rose from 2.2/10^5^ cases to 5.8/10^5^ cases, markedly higher compared to previous years [1]. Global incidence has been estimated to about 2.2 and 7.4 cases per 10^5^ at a global level. Mortality in these patients may be as high as 20%, and the majority of them will suffer from severe chronic pain or disability [19] (Table 1).

While it can be assumed that part of the documented increase must be attributable to the ageing population [20,21], at least in high-income countries, the availability of more modern diagnostic methods, especially medical imaging, have led to an increase in the number and accuracy of this diagnosis [22,23]. In parallel, newer epidemiological tools, including registry-based studies and real-world data analyses, are being increasingly used to monitor temporal trends and update the epidemiological profile of infectious spondylodiscitis [2]. Interestingly, a nationwide Japanese study found that admissions for pyogenic spondylodiscitis peaked in spring and were lowest in winter, whereas MRSA-associated cases did not show a significant seasonal pattern [24]. This lack of seasonality may suggest that MRSA spondylodiscitis is influenced less by climate-sensitive community transmission and more by year-round factors such as persistent colonization, healthcare-associated exposure, or bloodstream seeding; however, the underlying mechanism remains uncertain [24].

Regarding risk factors, in essence, every condition that is associated with immunosuppression is liable to lead to spondylodiscitis [3]. As such, immunosuppressive medications, chronic infections, substance abuse or misuse, and diabetes are implicated [19,25,26,27]. Regarding immunosuppressive medication, chronic steroid use is usually implicated; other groups at risk are patients with chronic renal failure and septicaemia with a different site of origin [28,29,30,31,32]. Male sex may also be considered a risk factor, as, in most studies, males appear to be affected almost twice as much, even though it is not known why this happens [33]. Curiously, this trend is not observed in pediatric spinal infections [34].

**Table 1:** Epidemiological data on infectious spondylodiscitis from different countries *.

Time Interval	Country	Incidence/Incidence Rise	Patient Age (Years)	Mortality (%)	Reference
1978–1982	Denmark	0.5/10^5^ cases	~60 (peak incidence)	n/a	[35]
1980–1990	Denmark	Increase (available as inc. rate in different age groups)	66 (median)	13	[5]
1987–1997	Norway	1/10^5^ cases	58 (mean)	n/a	[36]
1990–1995	Sweden	2.2/10^5^ cases	59 (median)	10	[37]
1995–2008	Denmark	2.2/10^5^ cases–5.8/10^5^ cases	66.6 (median)	n/a	[1]
2002–2003	France	2.4/10^5^ cases	59 (mean)	2.8–3.5	[38]
2007–2010	Japan	5.3/10^5^ cases to 7.4/10^5^ cases	n/a	6	[13]
2010–2019	France	6.1/10^5^ cases to 11.3/10^5^ cases	64.8 (mean)	7.3	[2]
2010–2019	South Korea	22.90/10^5^ cases to 35.79/10^5^ cases	58	n/a	[39]
2010–2020	Germany	10.4/10^5^ cases to 14.4/10^5^ cases	>70 (peak incidence)	4.56–6.47	[11]
2005–2021	Germany	5.4/10^5^ cases to 11/10^5^ cases	n/a	5.2	[18]
2012–2021	England	3/10^5^ cases to 4.4/10^5^ cases	63.5 (mean)	2–20	[17]

* For a certain country, data may be from specific cities or hospitals, and do not necessarily represent a general trend for the given time period; mortality refers to in-hospital mortality unless indicated otherwise.

## 3. Causes, Pathophysiology, and Clinical Manifestations

### 3.1. Causative Agents of Infectious Spondylodiscitis

In spondylodiscitis, the infection of the vertebral disc represents the primary event, and the neighbouring vertebrae and other spine elements represent secondary events [40,41]. Broadly speaking, spondylodiscitis can be divided into three different types, each caused by different pathogenic microorganisms (Table 2).

**Table 2:** Classification of infectious spondylodiscitis *.

Type	Subtype	Most Common Causative Agents	References
Pyogenic	Gram-positive bacteria-associated	*Staphylococcus aureus*	[42,43]
		*Staphylococcus epidermidis*	
		*Enterococcus faecalis*	
	Gram-negative bacteria-associated	*Escherichia coli*	[42,43]
Granulomatous	Tuberculous (Pott’s disease)	MTBC	[44]
	Brucellar	*Brucella melitensis* ^†^	[45,46]
	Fungal	*Candida* spp.; *Aspergillus* spp.	[47,48]
Parasitic		*Echinococcus granulosus*; *Schistosoma japonicum*, *S. mansoni*, and *S. hematobium*; *Taenia solium.*	[49,50]

* by type (based on the pathological aspect), and by subtype (based on the nature of the pathogen), along with the most common causative agents per category; ^†^ here we refer to classical brucellosis.

Bacteria are the main causative agents of spondylodiscitis, but fungi and parasites can also be isolated on occasion [51,52]. Though usually a single microorganism is behind it, polymicrobial presentation is also possible [42]. According to the data of the retrospective study conducted by Fröschen et al. [43], 120 distinct pathogens were found to be implicated in cases of spondylodiscitis, the vast majority of them—76.7% to be precise—being Gram-positive bacteria, with the most frequently encountered one being identified as *S. aureus*, at 26.6%. Coagulase-negative staphylococci accounted for 23.4% of the cases, mainly represented by *S. epidermidis* at 15%. This is also true of the findings of Doutchi et al. [42], where Gram-positive bacteria were present in 76% of cases, *S. aureus* being predominant, followed by *S. epidermidis*. *M. tuberculosis complex* was also isolated in some patients [42].

*Staphylococcus lugdunensis* is an increasingly recognized, though still uncommon, cause of invasive infection. It colonizes predominantly moist skin areas, especially the inguinal and perineal regions, and its clinical importance was likely underestimated in earlier years because isolates were often either misidentified or dismissed as contaminants. Improved species-level identification, particularly with matrix-assisted laser desorption/ionization time-of-flight mass spectrometry, has contributed to greater recognition of its true pathogenic role. While *S. lugdunensis* is most frequently associated with skin and soft tissue infection, bacteremia, endocarditis, and bone and joint infections are also well described. Within the musculoskeletal spectrum, it has been reported to affect prosthetic joints, native joints, and the vertebral column, with reviews suggesting that spondylodiscitis/vertebral osteomyelitis represents a meaningful, albeit still rare, manifestation of invasive disease. Vertebral infections due to *S. lugdunensis* are often associated with bacteremia and may follow a more virulent course than vertebral infections caused by other coagulase-negative staphylococci. Early reports described only isolated cases of vertebral osteomyelitis in immunocompetent hosts, underscoring its rarity, but more recent literature suggests that this entity may be underrecognized rather than truly exceptional [53,54,55].

Fungal spondylodiscitis is uncommon, but it should be considered in immunocompromised patients, in individuals with relevant travel or environmental exposure histories, and in cases with a subacute or indolent course that mimics tuberculosis or malignancy. In addition to the *Candida* and *Aspergillus* species, several endemic or opportunistic fungi have been implicated in vertebral infection, including *Cryptococcus neoformans/gattii*, *Coccidioides immitis/posadasii*, *Blastomyces dermatitidis/gilchristii*, *Histoplasma capsulatum*, and *Histoplasma duboisii*. Cryptococcal vertebral osteomyelitis is rare, but when skeletal disease occurs, vertebral involvement is among the most frequent osseous manifestations. It may present with lytic lesions and can closely resemble metastatic disease or tuberculous spondylitis [56]. Coccidioidal spinal infection is typically associated with dissemination from pulmonary disease acquired in endemic regions such as the southwestern United States, and although dissemination is rare, the vertebrae are reported to be the most common osseous site involved [56,57]. Blastomycotic spondylodiscitis is also rare, but it is well recognized and may involve the thoracic, thoracolumbar, or lumbar spine, often with paraspinal abscess formation and radiologic overlap with spinal tuberculosis [58]. Vertebral histoplasmosis due to *H. capsulatum* has been described both in immunocompromised and apparently immunocompetent hosts and may mimic neoplastic disease on imaging, further complicating diagnosis [59]. By contrast, *H. duboisii* spondylodiscitis is exceptionally rare, predominantly reported in sub-Saharan Africa, and is particularly important in the differential diagnosis of Pott’s disease because of its clinical and radiologic similarity to spinal tuberculosis. Overall, these fungal etiologies remain distinctly uncommon, yet they are clinically important because delayed recognition is frequent; diagnosis often requires biopsy with histopathology, culture, and sometimes molecular or antigen-based testing; and management may be prolonged and occasionally combined with surgical intervention when instability, deformity, abscess formation, or neurologic compromise are present [57].

Likewise, the findings of Taysi et al. [60] and Sheikh et al. [51] incriminate *S. aureus* at 27% and 28.5% of cases, respectively, with the latter also highlighting the pronounced involvement of *Mycobacterium* spp., namely *M. tuberculosis complex* at 42.9%, *M. abscessus* at 23.8%, and *M. chelonae* at 4.8% of cases. This pattern of notable pathogens is consistent with the results of Conti et al. [9], where although tuberculosis was identified substantially less, in only 10% of cases, the presence of *S. aureus* was significantly more prevalent, at 64%. Similarly, other researchers place the incidence of *S. aureus* at 55.1% [1] or even as high as 80% [61]. It is worth noting that tuberculous spondylodiscitis is one of the oldest human pathologies recognized in skeletal remains from osteoarcheological and paleoanthropological investigations [62,63,64,65,66,67].

### 3.2. Pathophysiology and Clinical Manifestations of Infectious Spondylodiscitis

Based on the localisation in the spine, spondylodiscitis can be classified as cervical, cervico-thoracic, thoracic, thoracolumbar, lumbar, lumbosacral, and sacral, while multiple foci have been described in some cases [11]. The majority of cases, based on the available literature, are found in the lumbosacral region, most probably due to its increased mobility and particular vascularisation characteristics; it is here that most cases of paravertebral abscesses as a complication also develop, while epidural abscesses are more frequent in the thoracic region [68].

Based on the aforementioned localisation, a preliminary differential diagnosis between the two forms of spondylodiscitis may be performed, as tuberculous spondylodiscitis preferentially affects the dorsal and lumbar spine, with the highest probability being the thoracolumbar junction; cervical and sacral infections are infrequent [69]. There is no unified typical clinical manifestation of spondylodiscitis, as patients may exhibit subclinical symptoms, such as mild pain, all the way to neurological deficits, signs of sepsis, and even death. Most manifestations are invariably associated with increased morbidity [2].

In the majority of cases, bacteria are spread hematogenously, having entered the bloodstream from different locations, like the oral cavity, skin, gastrointestinal system, etc. (Table 3). The arterial rather than the venous origin is more common. This can be explained by the presence of the bone marrow, which is associated with a voluminous blood supply of low velocity [70]. From a pathophysiological perspective, the mechanism of infection is different between adults and children, due to differences in the arterial supply of the discs and vertebrae.

In children, pathogens typically arrive via the persisting vascular channels in the disc space, while in adults, where the disc is avascular, the subchondral region adjacent to the disc is typically invaded by the pathogen. Arterial spread is the commonest route of infection in the case of tuberculous spondylodiscitis [71]. Regarding the venous route, rare as it is, Batson’s paravertebral venous plexus is implicated, especially in cases of sepsis in organs of the pelvic region [72], and the pre-vertebral pharyngeal venous plexus in cases of head and neck infections. In rare cases, it is possible for the pathogen to be directly delivered into the area from a nearby infected source, trauma, or iatrogenic inoculation.

**Table 3:** Different possible avenues of spinal infections, based on anatomical and systemic constraints.

Type of Dissemination	Avenue of Infection	Route	Comments	References
Hematogenous dissemination	Arterial spread	Pathogen arrival via persisting vertebral vascular channels	Children with bacteraemia	[15,73]
		Pathogen arrival in the subchondral region in adults	Adults with bacteraemia	[71,74]
	Venous spread	Pathogen arrival via Batson’s paravertebral venous plexus	Mostly in cases of pelvic organ sepsis	[72,74]
		Pathogen arrival via the pre-vertebral pharyngeal venous plexus	Uncommon but severe infections; primary infection usually in spaces inaccessible to clinical inspection	[74,75]
Direct extension	Different spread avenues depending on the primary source	Different depending on primary source	Patients with infections of the thorax, pelvis, head or neck, or of the psoas muscle	[74]
Trauma	Mechanical spread	Open or penetrating injury	Patients with large or deep wounds ± immunocompromised	[71,76]
Iatrogenic inoculation	Non-surgical methods	Diagnostic or therapeutic spinal punctures	Increased frequency in patients requiring frequent medical interventions	[71,74]
	Surgical methods	Spinal operations	Increased frequency due to more frequent and aggressive spinal operations	[71,77]

The onset of the condition is insidious, with the low specificity of the clinical manifestations hindering early diagnosis [52]. This is especially true if the focus of infection is in the thoracic region, where the disease may be completely paucisymptomatic; in the other regions of the spine, symptoms are usually observed at earlier stages [68].

The symptoms mostly associated with spondylodiscitis are back pain and fever, reaching 79% and 72% of cases, respectively [9]. This is in accordance with the findings of Taysi et al. [60], where the persistence or the residual character of the back pain was associated with adverse outcomes; in these patients, the presence of a sinus tract was also found to be notably higher. Radicular pain, when it appears, may complicate the diagnosis or even lead to unnecessary surgery [25,78,79,80]. Clinical manifestations do not seem to be different between the pyogenic and the tuberculous form, even though it has been noted that in the latter cases, the average duration of the symptoms is much longer [81,82]; spinal deformities, such as kyphosis, although having been noted in many spondylodiscitis patients regardless of etiology, are more common in the tuberculous group [81,83,84,85]. Dysphagia or torticollis may appear in patients with cervical spondylodiscitis, and paravertebral muscle spasms, if associated with spinal tenderness, the commonest sign, are characteristic [86,87,88]. A fluctuant mass instead of tenderness has been reported in some rare cases [89]. In children, as mentioned before, the presentation is quite diverse, with a variety of possible non-specific signs [90,91,92,93,94,95].

Moreover, while the pyogenic form usually affects two vertebrae, the tuberculous one is more likely to affect three; vertebral collapse and spinal deformity are also more frequent in the tuberculous group [81]. In the tuberculous form, it is more frequent to find a caudal extension, with multiple segments contiguously or intermittently affected; the subligamental spread of the infection is another defining characteristic [71]. In infectious spondylodiscitis, if more than one vertebra is involved, these are typically adjacent, which is expected as the intervertebral disc along with the adjacent parts of the upper and lower vertebrae are supplied by the same artery [96].

The severity of the condition lies in the serious complications associated with it, ranging from radicular nerve damage to abscess formation and endocarditis [9]. Permanent neurological impairment or even death can occur as a result [9,52,61], as previously mentioned. Neurological deficits can be recorded in as high as 47% [60] to 50% of patients [61]. These mainly include sensory deficit and motor weakness, but neurogenic bladder and loss of rectal tone have also been recorded [60]. Neurological damage is more commonly associated with cervical and thoracic abscess formation, compared to lumbosacral abscess formation [68]. Regarding spinal instability, this occurs more frequently in cases of lumbosacral infections, and mostly in younger individuals, as in the elderly, due to the comparatively lower physical demands, the preservation of spinal stability is much more likely [68].

In contrast to previous comparisons between culture-positive pyogenic spondylodiscitis and tuberculous spondylodiscitis, the findings of Kim et al. [97] suggest that fever, psoas abscesses, and paravertebral abscesses are more frequently encountered in patients with tuberculous etiology.

The results of McHenry et al. [98] are troubling as several of the patients passed away, more than one-third were left with residual disability, and 14% of them went through a relapse. This was often associated with extensive vertebral destruction and abscesses, post-surgical drainage, or debridement [98]. An independent association was also made between relapse and recurrent bacteremia, paravertebral abscesses, and chronically draining sinuses [98]. In Germany, after 2020, the mortality of admitted patients rose by 347% [18].

## 4. Diagnosis and Associated Challenges

The diagnosis of infectious spondylodiscitis is usually challenging on its own, and even in cases where there is a positive diagnosis, it is sometimes difficult to differentiate between its two forms due to their overlapping characteristics [9]. As with all spinal infections, a complete and thorough patient history is essential because it will determine the presence or absence of risk factors and also the potential avenues of infection. As many different systems as possible should be evaluated to determine if they are infected, and if they may be the primary locus of infection; a thorough neurological examination is also essential [99].

**Figure 2:**
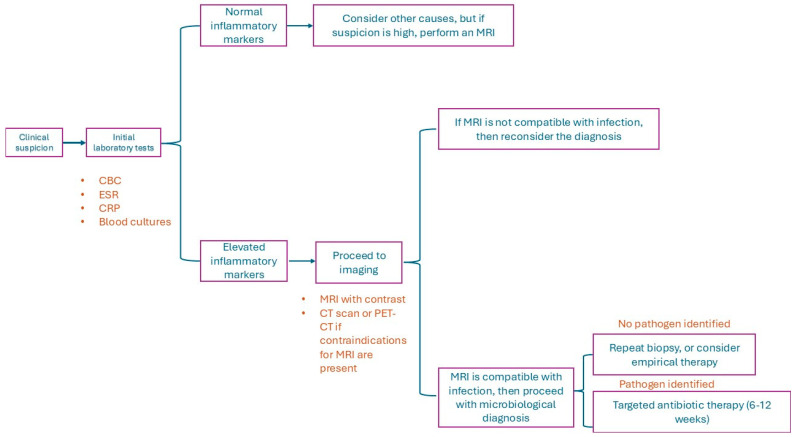
A diagnostic flowchart is useful for algorithmic decisions in spondylodiscitis. CBC = complete blood count, ESR = erythrocyte sedimentation rate, CRP = C-reactive protein, MRI = magnetic resonance imaging, CT = Computer Tomography, PET-CT = Positron Emission Tomography–Computer Tomography.

The diagnostic process ideally should comprise a combination of clinical, radiological, and microbiological evidence (Figure 2). Even though the initial presentation of patients usually does not point directly towards this pathology, a prompt diagnosis is essential, along with a determination of the pattern and location of infection, which also affect the course of treatment [99]. Ancillary instruments such as artificial intelligence applied to image analysis, predictive risk models, as well as quantitative metabolic imaging are increasingly used to support the diagnosis [100,101,102,103].

### 4.1. Imaging Diagnosis

Imaging is central to diagnosis, the assessment of disease extent, and the planning of surgical procedures. The main strengths and limitations for available modalities are presented in Table 4. It is generally customary to use both a sensitive and a specific method [71].

**Table 4:** Diagnostic methods for infectious spondylodiscitis: key findings, diagnostic performance, and major limitations.

Method	Sn	Sp	Common Findings	Challenges	References
**Radionuclide Imaging**					
Bone scintigraphy with Tc99m-biphosphonates	73–86%	31–69%	Characteristic triad of focal hyperperfusion, focal hyperaemia, and increased bony uptake	Decreased usefulness when there are underlying bone conditions or previous surgeries	[22,104,105]
^67^Ga	73–91% *	61–92% *	Increased concentration at infection sites	Increased time lag as imaging follows 18–72 h post-infection	[105,106]
Streptavidin/In111-biotin	93%	90%	Increased concentration at infection sites	An expensive method not available in most diagnostic facilities	[107,108]
Leucocyte labelling	Low	Low	Increased accumulation of leucocytes at infection sites	Successful imaging relies on the presence of intact chemotaxis; images in infectious spondylodiscitis patients resemble images of other pathologies; leucocytes sometimes fail to accumulate in the infected region	[109,110,111,112]
Ubiquicidin 29–41	100%	87.5%	Increased concentration at infection sites	An expensive method not available in most diagnostic facilities	[22,113]
**Ionizing Radiation Imaging**					
Plain radiography	30–65%	50–87.5%	Swelling of the soft tissues (if visible); destroyed fascial planes; periosteal reaction; osteopenia; cortical destruction at late stages	Not adequate visualization of soft tissues; the characteristic aspect of inflammation is not seen until 1–3 weeks after onset	[71,114,115]
CT	65–69%	95%	Pattern of bony destruction readily apparent; visualization of calcifications, bone fragments, or abscesses	Visualization of soft-tissue involvement is inferior compared to that of MRI; there may be a delay between symptom onset and characteristic aspects appearing on the CT	[99,116,117]
PET/CT	82–86%	95–100%	Higher focal FDG-18 uptake, especially in contiguous vertebrae	Presence of foreign bodies complicates diagnosis; inadequate visualization of nervous structures; increased cost and limited availability; high amount of irradiation for patients	[118,119,120,121,122,123,124]
**Magnetic Resonance Imaging**					
MRI	92%	96%	Hypointensity of the involved tissue in T1-weighted images; hyperintensity of the involved tissue in T2-weighted images; destruction of 2 or more adjacent vertebral bodies with disc involvement; epidural/paraspinal extension and abscess formation; narrowing of the infected disc(s)	Difficulty to reach a conclusive diagnosis before 35–50 days of infection; unavailable in many diagnostic facilities; limitations in patients with implants	[71,125,126]
**Molecular Diagnostic Methods** ^†^					
PCR	Variable (42.9%)	Generally high for pathogen identification, but dependent on contamination control and reference standard (100%)	Detection of bacterial DNA directly from biopsy or other clinical specimens; useful particularly in culture-negative cases	Detects bacteria but not fungi; does not reliably distinguish contamination, colonization, or clinically irrelevant DNA; reduced sensitivity in low-burden samples; performance is affected by prior antibiotics; may not differentiate closely related *Mycobacterium* species without additional targets or sequencing; usually adjunctive rather than first-line testing	[127,128,129]
mNGS	Variable (72.7–80%)	Variable; often lower clinical specificity than analytical specificity (93.3–100%)	Unbiased detection of microbial nucleic acids from clinical samples, including potential identification of unexpected or fastidious pathogens	Expensive and not widely available; clinical specificity may be limited because commensal, contaminant, or non-viable microbial DNA may be detected; interpretation requires close clinicopathological correlation; does not inherently distinguish infection from colonization; fungal detection is possible only if the platform and bioinformatic workflow are optimized for fungi; standardization remains limited	[130,131,132]

* When combined with SPECT. ^†^ For molecular methods, sensitivity and specificity refer to pathogen detection/identification rather than to the diagnosis of infectious spondylodiscitis itself. Reported performance varies according to specimen type, pathogen burden, prior antimicrobial exposure, target selection, laboratory workflow, and reference standard. Broad-range bacterial 16S rRNA PCR detects bacterial DNA only and does not identify fungal pathogens; fungal detection requires ITS-based sequencing/PCR or other dedicated fungal assays. Molecular testing may be performed on biopsy material and, in selected settings, on blood specimens, particularly when a biopsy is not feasible, although diagnostic yield is generally lower in blood for localized spinal infection. CT = computed tomography, PET = positron emission tomography, MRI = magnetic resonance imaging, PCR = polymerase chain reaction, mNGS = metagenomic next generation sequencing, Sn = sensitivity, Sp = specificity.

Imaging findings should be correlated with clinical data and interpreted in the context of several important degenerative, inflammatory, traumatic, or metabolic mimics and coexisting factors [71,133,134,135,136,137,138,139,140] (Table 5). Even after the exclusion of potential differential diagnoses, a combination of clinical and paraclinical investigations should be used to differentiate between pyogenic and tuberculous spondylodiscitis (Table 6).

**Table 5:** Most probable differential diagnosis in infectious spondylodiscitis patients, listed in alphabetical order.

Differential Diagnosis	Type of Pathology	Similar Features	Differentiating Features	References
**Non-septic Inflammatory Conditions**				
Andersson lesion (Ankylosing spondylitis)	Manifestations of aseptic spondylodiscitis or of advanced ankylosing spondylitis; most common in patients with ankylosing spondylitis	Central and peripheral focal erosions at the level of the thoracolumbar junction or lumbar spine, involving more than one vertebra; sclerosis of the end plates; marked bone edema	No epidural or paraspinal spread; often in association with manifestations of spondylarthritis	[124,141,142,143,144]
Neuropathic spinal arthropathy (Charcot spinal arthropathy)	A rare progressive osseous and ligamentous injury leading to the deterioration of vertebral joints	Bone erosion and destruction; bone marrow edema with possible spread in the surrounding tissues	Presence of the vacuum disc phenomenon; presence of osseous debris; joint dislocation	[124,137,145]
SAPHO syndrome with spinal involvement	An umbrella term covering a variety of osteoarticular and dermatological manifestations	Lesions mostly on the anterior side of lumbar and/or lumbar vertebrae; erosions of vertebral corners; bone marrow edema	Involvement of extraspinal joints, along with osteosclerosis and hyperostosis	[124,141,142,146,147,148]
**Metabolic Conditions**				
Destructive spondyloarthropathy	A serious complication of long-term haemodialysis due to amyloid deposition in the spine	Usual involvement of the discs and posterior spinal elements of the lower spine; marked narrowing of disc space, subchondral erosion, and end plate cysts	Differential diagnosis is based on patients belonging to the particular risk group, and by using specific MRI techniques	[124,149,150,151]
Micro-crystalline spondylitis	Deposits of hydroxyapatite and calcium pyrophosphate inside the intervertebral discs	Vertebral body edema, and inflammatory reaction of the surrounding tissues; intra-discal abnormal signal intensities at MRI	CT of the affected regions will reveal crystals of increased density	[124,152,153]
**Degenerative Conditions**				
Modic type 1 lesion	Presence of edema and inflammatory changes in the bone beneath the endplate	Irregular end plate contours; possible subchondral cysts; vertebral edema	Extended disc thinning with low T2 signal intensity; end plate borders usually spared; association with other Modic lesions; presence of the claw sign	[124,154,155,156,157]
Erosive osteochondritis	A destructive form of intervertebral osteochondrosis with inflammatory degeneration of the intervertebral disc	Disc degeneration with widespread erosion of the end plates	Relatively preserved intranuclear cleft on T2-weighted SE images; limited bone marrow damage and hyperaemia; lack of paraspinal inflammation	[71,139,158]
Schmorl’s nodes	Herniation of the nucleus pulposus through the cartilaginous and bony end plate into the body of the adjacent vertebra	Narrowing of the intervertebral disc space and poor definition of end plates	No pronounced changes in bone marrow signal intensity at MRI; high T2 intensity of the disc space in T2-weighted images	[71,134,159]
**Traumatic Conditions**				
Osteoporotic fractures	Fractures due to compromised bone density due to metabolic causes	Collapse of the endplate and paraspinal fluid collection	Low-signal intensity rim lining parallel to the endplate, and no disc involvement; easy differentiation at MRI	[141,144,160,161]

**Table 6:** Basic elements for differential diagnosis between the pyogenic and tuberculous forms of infectious spondylodiscitis.

Categories	Pyogenic Form	Tuberculous Form	References
Symptom duration (onset)	3 months (acute or subacute)	7 months (subacute)	[81]
Patient history	Recent surgery, bacterial infection, immunosuppressed status, or IV access device use	A history of tuberculosis, or symptoms suggestive of tuberculosis (either spinal or extraspinal), and/or residence/visit in a country where tuberculosis is endemic	[124,162]
Signs of sepsis	More common	Less common	[162]
High fever	More common	Less common	[162]
Spinal deformity	Less common	More common	[162]
Paraclinical investigations	Markers of inflammation are usually raised	Markers of inflammation may be slightly raised or even normal	[162]
X-ray	Osteopaenic changes in the subchondral layer; erosive and blurred endplate margins, diminished intervertebral spaces; paravertebral soft tissue mass	Rarefaction of vertebral end plates, osseous destruction, disc height loss, new bone formation, and soft tissue abscesses, along with gibbous deformity	[124,163]
CT	Bone destruction and involvement of paravertebral tissue, along with abscesses or gas inclusions; no gibbous deformity and calcified masses	Visualization of the characteristic gibbous deformity and bone destruction; loss of cortical definition; presence of calcified and large paraspinal masses	[124]
PET/CT	Increased uptake value in the infected region	Maximum standardized uptake value is higher if the imaging is not delayed	[164,165]
MRI	Less pronounced T2-hypointensity in some patients; involvement of 2 vertebrae is more frequent; lower degree of bone invasion; decreased loss of cortical definition; diffuse and homogenous enhancement pattern; presence of a disc abscess with peridiscal rim is more common	More pronounced T2-hypointensity in some patients; Involvement of 3 vertebrae is more frequent; higher degree of disc preservation; increased loss of cortical definition; focal and heterogenous enhancement pattern; frequent vertebral collapse and spinal deformity; absence of osteosclerosis and rim-like peripheral contrast enhancement with sharp demarcation on T1-weighted images with specific protocols	[71,81,166,167,168]

#### 4.1.1. Radionuclide Imaging

Historically, bone scintigraphy had the highest sensitivity for infectious spondylodiscitis until the introduction of MRI [71]. The basic method is the use of Tc4399m-biphosphonates, whose uptake depends on vascularisation and new bone formation. In patients suspected of having osteomyelitis, a triphasic bone scan can be performed, comprising the perfusion phase with dynamic images, the tissue phase with static imaging, and the skeletal phase with static images 2–4 h post-administration. The typical presentation of infectious spondylodiscitis is focal hyperperfusion, focal hyperaemia, and bony uptake [22]. A major shortcoming of this method is its loss of specificity in cases where pre-existing conditions affect the bone [112]. There have been many different research efforts on quantifying the specificity, sensitivity, and accuracy of the methods, but there is no consensus on these parameters [80,104,169]. Methods using Tc-labelling have also been developed for fungal infections [170].

Another possibility is the use of Ga3167, which, in the human body, is transferrin-bound or lactoferrin-bound, and as such concentrates in areas with increased blood flow, such as sites of infection. Part of the gallium is also directly uptaken by bacteria, hence contributing to its concentration at infection sites [112]. This method is useful [171], especially if combined with SPECT [106,107,169,172,173,174,175].

Yet another radionuclide used is In49111, which is bound to biotin, i.e., vitamin B7, which participates in glucose metabolism regulation and pancreatic β-cell function [22]; some bacteria also use biotin as a growth factor [176,177]. Imaging is performed in two steps with streptavidin/In49111-biotin, and it is quite accurate as streptavidin accumulates in infection sites due to increased vascular permeability, along with In-biotin. This complex has high affinity for both inflammatory regions and neoplastic tissues, hence it is a very sensitive and specific method with high accuracy [107,108,178]. Interestingly, and this is important for patients already under treatment for suspected infectious spondylodiscitis, or some other condition, antibiotic administration does not appear to have any effect on the accuracy of the test [178]. Other advantages of this method include minimal bone marrow uptake and less patient irradiation, compared to other radionuclide methods, along with rapid imaging [22].

Leukocyte labelling has also been tried, with either oxyquinolone or exametazime, themselves labelled with In49111 or Tc4399m respectively; the uptake of these labelled leukocytes depends on a number of different factors, not least the type and number of leukocytes being labelled; since most of them are neutrophils, it follows that the method will be the most sensitive for neutrophil-mediated infections [112]. The presence of intact chemotaxis will also influence the accuracy of the results [22]. While, in theory, this method should be of diagnostic usefulness, in about half of the cases, labelled leukocytes do not appear to concentrate in bone infection sites [109], although why this happens is not known; in fact, marrow uptake may be increased in the healthy bone. Patients with shorter symptom duration apparently have higher uptake at infection sites. This test, even if positive, can point towards other pathological bone conditions, such as neoplasias, fractures, and Paget’s disease [109,111,112].

Finally, research has been carried out using labelled antimicrobial peptides, whose expression is either constant or microbe-induced [179,180]. The team of Dillmann-Arroyo et al. [113] studied Tc4399m-labelled ubiquicidin, in patients suspected of having infectious spondylodiscitis, and reported high sensitivity and specificity values.

#### 4.1.2. Plain Radiography

Even though a simple X-ray is a simple, cheap, and rapid diagnostic modality, available in most healthcare settings, changes in the affected region will appear from one to three weeks post-infection [71]. For a change in bone density to be accurately detectable in plain radiography, there would have to be a reduction in bone density of about 30% to 40% [7,181,182,183]; for this to occur in infectious spondylodiscitis, the infection must be ongoing for 2–8 weeks, after initial symptom manifestation [124].

**Figure 3:**
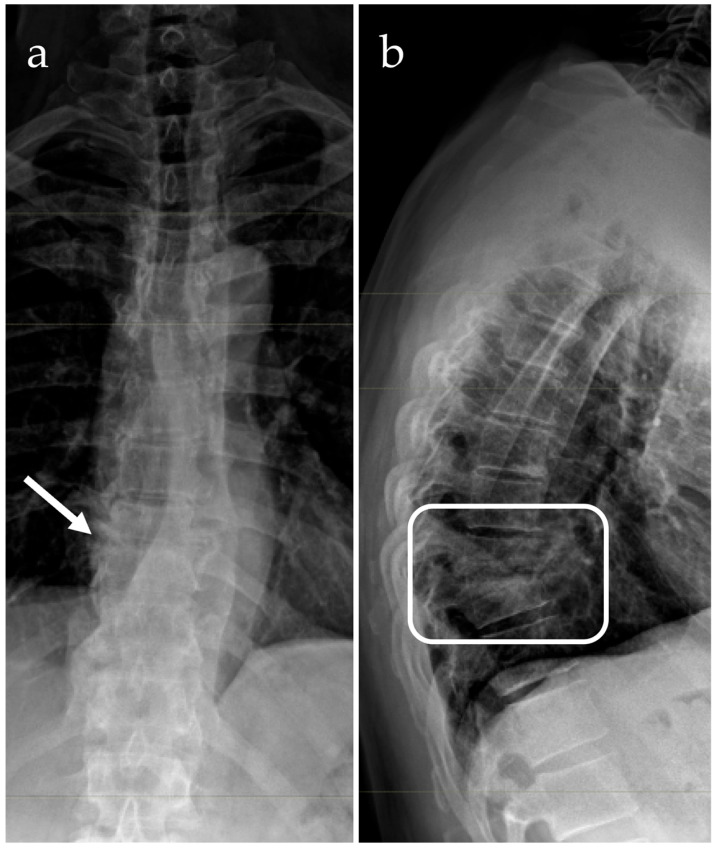
A plain X-ray of the thoracic spine in a male patient with bacterial spondylodiscitis, antero-posterior (**a**) and lateral (**b**) view. Mild T9 and T10 vertebral collapse and reduced disc height (arrow in (**a**) and white case in (**b**)).

The relevant radiographic signs are a decreased density within the subchondral region—therefore, this part is more radiolucent—usually in the anterior part, along with a narrowing of the intervertebral disc space and loss of the clear end plate margins [71]. A gradual loss of margins in the opposite end plate and an increase in radiolucency in the vertebra is associated with a late infection stage, as inflammation spreads and bone destruction progresses. Due to the high radiolucency of soft tissues, their displacement due to inflammation may be difficult to visualize; typically, for the X-ray to have a characteristic aspect of infectious spondylodiscitis, the disease must be in a fairly advanced stage [71]. In general, there is no consensus as to the potency of plain radiographs to reveal evidence of abnormalities in such patients, with evidence ranging from 58% to 89% of cases [19,22,184]. Only for spinal tuberculosis of the upper lumbar and thoracic spine are plain X-rays the primary diagnostic tool [185] (Figure 3).

#### 4.1.3. CT

As a corollary of its vastly superior contrast resolution for soft tissues, CT has a far higher sensitivity for the diagnosis of infectious spondylodiscitis. As a rule, in all spinal infections, abnormalities at the level of the spine appear earlier in CTs compared to X-rays [99]. Under certain circumstances, they may be the only option available, other than plain radiographs and bone scintigraphy, if there are implanted devices in the patients [22]. The use of contrast agents can be considered to further enhance their imaging capacity, specifically for the epidural venous plexus [163].

The reduction in density of the intervertebral disc can be visualized in very early stages, as well as the thinning of the paravertebral fat. Bone destruction and sequestra formation can be seen in later stages [140]. Moreover, the extension of the inflammation can be seen clearly, along with potential abscesses and epidural phlegmons, causing spinal cord compression [71]. CT is superior even to MRI in visualizing both the sequestra and the subsequent calcifications; the latter points towards tuberculous spondylodiscitis. CTs are also useful for guided biopsies, which are usually crucial in establishing the cause of the infection [71,99].

#### 4.1.4. PET

Compared to labelled compound administration, PET imaging is inherently of higher resolution and permits a more exact localisation of the lesion focus [106,186]. A semi-quantitative analysis of uptake is easier, thus permitting the differentiation between non-infectious and infectious sites [22].

The most-used positron-emitting agent is fluorine-18-fluorodeoxyglucose, or FDG-18, whose cellular uptake is mediated by a host of different mechanisms [187,188,189]. In infection, its uptake at the infection sites is upregulated, due to different reasons which have been explored in detail in the relevant scientific literature [190,191,192]. Specifically in the case of infectious spondylodiscitis, there have been a number of investigations [186,193]. Accuracy seems to be as high as 100% in certain cases [110].

PET/CT has lower accuracy if foreign objects, such as surgical prostheses, exist in the area [118,121,122]. Compared to MRI, PET/CT may be slightly superior in diagnosing early lesions [106]; in other studies, it has proven to be of equal diagnostic effectiveness or even greater [119,174,194,195,196,197,198,199,200,201].

Another PET modality is the employment of ^68^Ga bound to citrate, which is superior to ^67^Ga, in several ways. Relatively little research has been undertaken on the role of this radiotracer in the diagnosis of infectious spondylodiscitis. The sensitivity, specificity, and accuracy values are similarly high to that of FDG-18 [202]. Other substrates for ^68^Ga to bind have also been researched [203]. Irrespective of the particular agent used, PET or PET/CT remains a viable and useful alternative, especially in cases where MRI cannot be performed [22].

#### 4.1.5. Magnetic Resonance Imaging (MRI)

This is the gold standard in imaging diagnosis of infectious spondylodiscitis, with both sensitivity and specificity being over 90%; a major constraint is that to achieve such a good diagnostic accuracy, the infection must be ongoing for over 30 days [204]. If performed with paramagnetic contrast agents, and specifically fat-suppression techniques, MRI combines the sensitivity of scintigraphy and the specificity of CT or plain radiography, in advanced stages [205,206,207,208].

**Figure 4:**
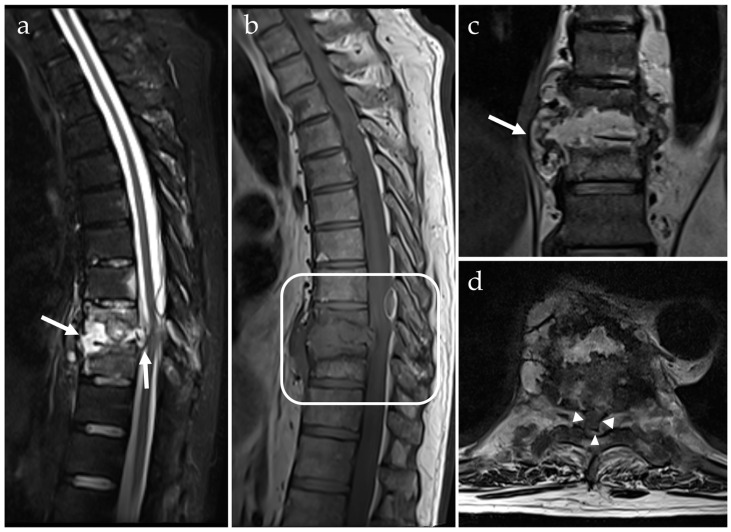
MR examination (sagittal STIR—(**a**); sagittal T1-weighted image—(**b**); coronal T2-weighted image—(**c**); axial T2-weighted image—(**d**)), depicting spondylodiscitis at the level of T9–T10: osteolysis and destruction of the intervertebral disc, with perivertebral and epidural abscesses (high signal intensity on T2/STIR and low signal intensity on T1—arrows), compressing the spinal cord (arrowheads). White case indicates the level affected by spondylodiscitis.

For the early stages of infection, the short tau inversion recovery (STIR) sequence is the most sensitive method; with the addition of a T1 spin-echo (SE) sequence, both before and after Gd contrast administration, it is possible to demonstrate clearly all relevant anatomical details, and distinguish between necrotic, non-necrotic, and inflammatory components [209,210] (Figure 4). A detailed description of the appearance of the infected area, using different MRI protocols and depending on the stage of the infection, is provided by Jevtic [71]. Disease progression, evidenced by a reduction in bone marrow edema and an increase in fibrovascular tissue, and response to treatment can also be monitored using MRI [71].

It is even possible to perform a differential diagnosis between the two forms of the disease in certain cases. Several differences exist, namely, a difference in signal intensity in the T2-weighted MRI, a difference in the grade of bone destruction, a loss of cortical definition, different enhancement patterns of the vertebral bodies, and a difference in the regional and extension characteristics of abscess patterns [81], among others. The differences between the imaging findings in cases of pyogenic and tuberculous spondylitis have been discussed by a number of researchers, and certain diagnostic clues may be provided by MRI [81,167,208,211,212,213,214,215,216], although obtaining the culture is still the gold standard of diagnosis. MRI may not be particularly useful in monitoring improvement and response to treatment [217,218]; an MRI follow-up is required only in cases where there is no clinical improvement 4 to 6 weeks post-treatment [22].

### 4.2. Paraclinical Diagnosis

Regarding paraclinical diagnosis methods, the selection and interpretation of the results are usually not straightforward, as it has to be appreciated that most patients present with vague symptoms of neck and back pain, along with some feeling of malaise [99]. A usual sign of infectious spondylodiscitis, in its pyogenic form at least, is focal tenderness, which, combined with radiological data and paraclinical investigations (Table 7), can point towards the right diagnosis.

**Table 7:** Basic laboratory investigations with abnormal levels/results in patients with infectious spondylodiscitis.

Marker Category	Parameter	Values	Remarks	References
Complete blood count (CBC)	Haematocrit	Decreased or normal	Pathological levels may appear in different percentages depending on sex	[37]
	Red blood cells	Decreased or normal		
	Leukocytes	Normal or elevated (less than 45% of patients)	A normal leukocyte count does not exclude the possibility of infection; usually normal in immunocompromised or elderly patients; neutrophils may be increased in pyogenic forms	[99,162]
	Platelets	Normal or increased (in about half of the patients)	Even if elevated, the elevation may not be very pronounced	[37]
Inflammatory markers	CRP	Elevated	Most patients presenting with symptoms will have an elevated CRP; can be used to monitor treatment effectiveness	[162,184,219]
	ESR	Usually elevated (in about 75–95% of cases)	Sensitive marker for inflammation/infection, but not specific; can potentially be used as a prognostic marker	[162,220]
Enzymatic assays	ALP	Elevated (about 50% of patients)	Useful when interpreted in conjunction with other paraclinical investigations and clinical data	[84]

CRP = C-reactive protein, ESR = erythrocyte sedimentation rate, ALP = alkaline phosphatase.

#### 4.2.1. Indicative Blood and Serum Markers

Note that the C-reactive protein (CRP) is generally more useful than the erythrocyte sedimentation rate (ESR), given that it has a more rapid time of normalization, and can therefore be used to also monitor treatment effectiveness [184], and should have dropped to normal levels 3 months post-successful treatment [219]. Its levels are increased in most patients presenting with symptoms [52,162].

In the majority of infectious spondylodiscitis patients, the ESR is increased [36,41,87,88,89,184,220,221,222] even though this may not be helpful in the presence of a primary infection focus. While the rise in ESR is indicative if present, its levels do not correlate to disease severity; on the other hand, it is believed that it may have a prognostic value [220]. A general marker for surgical site infections, procalcitonin, may or may not be helpful, based on different researchers [52,223,224].

A high leukocyte count remains a characteristic of infectious spondylodiscitis and is more common in the pyogenic form [225]. Even though an increased leucocyte count may be seen, other biomarkers may prove more useful. While there is no consensus in the scientific literature about the percentage of patients in whom this marker is elevated, it ranges somewhere between 33% to about half of patients in whom analyses have been performed [36,37,41,87,88,89,184,220,221,226,227,228]. It is usually not elevated in immunocompromised patients [229] even though another study disagreed with this assertion [230]. When comparing patients with pyogenic and tubercular or brucellar spondylitis, neutrophil count was found to be more elevated in those with the pyogenic form [84,231].

Regarding other blood and serum tests, about 70% of patients may be anemic, and the alkaline phosphatase value may be increased in associated vertebral collapse [37,41,84,228]. In general, platelet count increases with inflammation [232], but this is not typical for all patients [37]. Finally, when risk factors are present, a wide variety of interferon-γ release assay (IGRA) tests can be considered, and they provide superior diagnostic performance to tuberculin skin tests [99,233].

#### 4.2.2. Pathogen Identification Methods

After confirming the diagnosis of infectious spondylodiscitis, it is essential to identify the causative agent. Even though, as mentioned before, it is essential to start with an empirical antibiotic scheme as quickly as possible, a blood sample for an aerobic and an anaerobic culture should ideally be obtained before any drug administration. This will hopefully lead to pathogen identification [99]; even so, it must be remembered that in most cases, pathogen identification via cultures remains difficult, with a success rate of around 30% and rarely rising up to 70% [99,125]. Polymicrobial infections are even more difficult to identify, using standard microbiological techniques alone [43,221].

The diagnostic value of the CT-guided spinal biopsy has been studied by different authors. The diagnostic performance of CT-guided spinal biopsy is described as high as 75.8% for tuberculous spondylodiscitis, including histopathological examination, and as high as 41% for pyogenic spondylodiscitis A CT-guided spinal biopsy should be obtained whenever it is possible, as it has a low rate of complications, but can add important data to the diagnostic and treatment algorithm [234]. The blend between microbiological and histopathological examination following a biopsy has proven to have around 60–65% sensitivity, and up to 100% specificity in etiological diagnosis [235].

In case there are no severe signs present, such as sepsis or a significant neurologic deficit, a second biopsy can be attempted, which can elevate the microbiological diagnosis to 60–80% chances of a positive result [236,237]. In the case of negative results, it is advised to perform molecular biology techniques and serology for intracellular bacteria, or mycobacteria [235].

In endemic areas, blood cultures should be obtained and appropriate serological testing for *Brucella* spp. should be performed when brucellar spondylodiscitis is suspected. In laboratories still using manual blood culture methods, prolonged incubation or specific communication with the microbiology laboratory may be required to optimize recovery. Fungal blood cultures may also be considered when clinically indicated. In patients with risk factors for tuberculosis or with suggestive imaging findings, a purified protein derivative test and an interferon-γ release assay may provide supportive evidence [129]. If blood cultures and serological investigations remain negative, CT- or ultrasound-guided biopsy should be considered to obtain material directly from the site of infection. Although a percutaneous biopsy is generally safe, the amount of tissue obtained is often limited, and microbiological yield remains modest, with pathogen identification in only about 30% of cases [238]. Thus, in the majority of cases, a second, more invasive biopsy method may be needed for additional testing [52].

In the last decade, the advent of molecular diagnostic methods has also helped towards accurate and rapid pathogen identification. Polymerase chain reaction (PCR) has been used to detect specific parts of the microbial genome. Molecular techniques like 16S rRNA have been found to be superior to standard cultures in identifying the nature of the pathogen. Given its broad-spectrum capacity, it should be used to supplement standard microbiological methods, when the results are ambiguous, or when all cultures turn back negative [127,128,129]. However, PCR-based methods have important limitations, including the inability to provide phenotypic antimicrobial susceptibility testing, the risk of contamination, reduced sensitivity in low-burden specimens, and when broad-range 16S rRNA assays are used, the inability to detect fungi or reliably differentiate closely related mycobacterial species without additional targeted assays or sequencing [239]. However the gold standard for *M. tuberculosis* complex diagnosis remains culture identification completed with nucleic acid amplification tests, which are able to identify MDR and rifampicin-resistant strains [240].

A more recent method, metagenomic next-generation sequencing (mNGS), may be employed, offering rapid and accurate detection, but its high cost limits its current availability as a mainstay of diagnosis [132]. Additional limitations of mNGS include imperfect clinical specificity—because detected nucleic acids may reflect contamination, colonization, transient translocation, or non-viable organisms rather than true infection—and the lack of phenotypic antimicrobial susceptibility testing [241]. Some laboratories now report antimicrobial resistance markers from mNGS, which may provide useful adjunctive information, especially in culture-negative or non-viable infections and when blood is used because a biopsy is not feasible, although this remains inferior to conventional phenotypic antimicrobial susceptibility testing and requires cautious clinical interpretation [242].

#### 4.2.3. Histopathological Diagnosis

Histopathology is extremely valuable in low microbiological yields, in suspicion of tuberculosis infection, or when more information on tissue architecture can guide the diagnosis [84,162,243,244,245]. Detection rates are much higher, starting from around 70% and rising up to over 90%, based on available data [246]. Guided biopsies provide histopatological samples, but may increase the risk for epidural abscess formation in certain cases [99].

The histological findings in patients with pyogenic spondylodiscitis may have a range of different appearances, usually revealing a chronic inflammatory cell infiltrate, comprising lymphocytes, macrophages, and plasma cells. Usually, a characteristic, increased polymorphic neutrophil infiltrate can be evidenced. Other than that, and depending on the severity of the disease and the time post-infection of the biopsy, different amounts of osseous, fibrous, cartilaginous, and fat tissue will typically be seen [247].

Tuberculous spondylodiscitis typically appears with tubercles with central caseating necrosis; cold abscesses may develop in the surrounding tissues, and at later stages, there may be fibrosis and calcification as the caseating lesions are being replaced [71]. At any rate, the presence of granulation tissue points towards tuberculosis or, more rarely, brucellosis [99]. When considering that in a number of cases, even from samples from biopsies, no microorganism can be cultured, the absence of polymorphic neutrophil infiltrates and/or the presence of caseating necrosis may be the only factors indicating the tuberculous nature of the disease [238,248].

Acid-fast bacilli (AFB) staining is a histopathological and microbiological technique used to detect organisms with mycolic acid-rich cell walls that resist decolorization by acid-alcohol, most notably MTBC. In tissue sections or smear preparations, Ziehl–Neelsen staining remains the classical method, while auramine-based fluorochrome staining may offer higher sensitivity in paucibacillary specimens. A positive AFB stain supports the presence of mycobacterial infection, but the finding is not specific for *M. tuberculosis*, as other acid-fast organisms, including non-tuberculous mycobacteria and *Nocardia* spp., may also stain positive. Conversely, a negative AFB stain does not exclude tuberculous spondylodiscitis, particularly in specimens with low bacillary burden. Therefore, AFB staining should be interpreted in conjunction with histopathological findings, culture, molecular assays, and the clinical-radiological context [249].

## 5. Treatment

In most cases, the treatment is complicated both by the diagnostic delay and the variability of the causative pathogens, for even if the disease itself is identified, it can be challenging to determine the exact pathogen. Comorbidities such as haemodialysis may also complicate the treatment and influence the outcome [250].

While it is possible to start the administration of broad-spectrum antibiotics in the absence of a positive identification, targeted treatments offer the best chance of success; acting on multiple pathogenic pathways can improve antibiotic effectiveness [251,252,253]. Even so, since identification of the pathogen is achieved in roughly 2/3 of cases, empirical treatment is necessarily used in the rest of the cases. If global signs of sepsis are present, the treatment must be initiated immediately; on the other hand, if no primary infection focus can be identified, a transoesophageal ultrasound may be necessary to rule out bacterial endocarditis [99], which, if present, complicates the treatment.

### 5.1. Antibiotic Treatment: Constraints and Challenges

Even though guidelines for surgical interventions exist, each patient must be examined and assessed separately. If there are no neurological deficits or instability, and no previous failed therapy, a conservative approach can be attempted [99]. Depending on the particular pathogen detected, the antibiotic regimen is adjusted accordingly (Table 8), although the emergence of resistance strains may complicate matters.

**Table 8:** The most common pathogens identified in infectious spondylodiscitis patients, the associated treatment schemes, and challenges; a comprehensive list of pathogens is provided by Zou et al. [52].

Family	Genus	Species	Recommended Treatment	Comments	References
**Gram-positive bacteria**					
*Actinomycetaceae*	*Schaalia*	*S. odontolytica*	Penicillin or amoxycillin	Cases of systemic infections are very rare but may require prolonged treatment schemes	[254]
*Corynebacteriaceae*	*Corynebacterium*	*C. amycolatum*	Penicillin ± ceftriaxone ± metronidazole (or other appropriate antimicrobial agents)	Little is known about resistance patterns; biofilm formation in artificial surfaces may complicate treatment	[255,256,257]
		*C. striatum*	Vancomycin or linezolid as the main targeted agents; daptomycin as an alternative when susceptibility is confirmed	Corynebacterium striatum is a rare but increasingly recognized cause of invasive spinal infection and should not automatically be dismissed as a contaminant, particularly when isolated from blood cultures and deep biopsy specimens. Management can be challenging because susceptibility profiles are variable and multi-drug resistance is increasingly reported	[258]
*Enterococcaceae*	*Enterococcus*	*E. faecalis*	Amoxicillin ± gentamicin	Fairly low resistance profiles in most countries; preventive strategies are needed to prevent resistance emergence; nosocomial pathogens are more resistance	[162,259,260]
		*E. faecium*	Vancomycin ± gentamicin	Resistance profiles have been noted in nosocomial settings; otherwise, resistant strains are not overly prevalent	[162,261,262]
*Micrococcaceae*	*Micrococcus*	*M. luteus*	Linezolid or erythromycin	Broad resistance spectrum to cephalosporins or quinolones reported from some strains	[263]
*Peptoniphilaceae*	*Parvimonas*	*P. micra*	Ampicillin, clindamycin or penicillin (or other antibiotic as determined by susceptibility testing)	A very rare pathogen, mostly identified in patients with malignancies, diabetes mellitus or post-arthroplasty; antibiotic treatment in infectious spondylodiscitis varies from patient to patient	[264,265]
*Propionibacteriaceae*	*Cutibacterium*	*C. acnes*	Doxycycline, tetracycline, or clindamycin	Some resistant strains have been recorded, although resistance to conventional antibiotic agents is relatively rare; biofilm formation may complicate treatment	[266,267]
*Staphylococcaceae*	*Staphylococcus*	MSSA	Flucloxacillin, penicillin, or ceftriaxone	Usually, there is resistance to β-lactams even in MSSA strains	[162,268]
		MRSA	Vancomycin or teicoplanin	MRSA and multi-drug resistant strains are mostly encountered in nosocomial settings	[162,269]
*Streptococcaceae*	*Streptococcus*	*S. agalactiae*	Penicillin or ceftriaxone	Increasingly resistant strains have been reported in patients from certain countries; biofilm formation accounts for prolonged persistence in many cases	[162,270,271]
		*S. oralis*			
**Gram-negative bacteria**					
*Brucellaceae*	*Brucella*	*B. melitensis*	Doxycycline + streptomycin, or doxycycline + rifampicin	Resistance of B. melitensis to common therapeutic agents surpassing 70% in certain cases	[162,272,273]
*Enterobacteriaceae*	*Enterobacter*	*E. cloacae*	Carbapenems, or cefoperazone/sulbactam, or piperacillin/tazobactam, or cefepime	Rising resistance to β-lactam antibiotics and carbapenem antibiotics, and other antibiotic classes	[274,275]
	*Escherichia*	*E. coli*	Prolonged meropenem infusion	Persistently high resistance to a number of mainstream antibiotics	[276,277]
	*Klebsiella*	*K. pneumoniae*	Cephalosporin ± aminoglycoside, or quinolone ± aminoglycoside	Resistance patterns of different strains to different antibiotics have been identified; biofilm formation complicates treatment	[278,279]
	*Salmonella*	*Salmonella* spp.	Culture-guided antibiotic therapy, most commonly third-generation cephalosporin (ceftriaxone) or a fluoroquinolone (ciprofloxacin) or ampicillin and chloramphenicol	*Salmonella* spondylodiscitis is rare and may closely mimic spinal tuberculosis. Gastrointestinal symptoms may be absent, so tissue, pus, bone, or blood culture is essential for confirmation. Surgical intervention may be needed in selected cases with osseous instability, abscess formation, pain refractory to medical therapy, or neurologic deficit	[280]
*Morganellaceae*	*Proteus*	*P. mirabilis*	β-lactams, aminoglycosides, fluoroquinolones, or trimethoprim-sulfamethoxazole	Multiple virulence factors, biofilm formation, and resistance patterns complicate systemic infections	[281,282,283]
*Prevotellaceae*	*Prevotella*	*P. intermedia*	Amoxicillin/clavulanic acid, metronidazole, tetracycline, or clindamycin	A rare cause of systemic infections or infections in sites other than the oral cavity; sometimes difficult to suspect or detect	[284,285]
*Pseudomonadaceae*	*Pseudomonas*	*P. aeruginosa*	Ceftazidime ± aminoglycosides, meropenem ± aminoglycosides, or ciprofloxacin	Multi-drug resistant strains necessitate new treatment strategies; different treatment schemes can be used to address resistant strain infections	[162,286,287]
*Yersiniaceae*	*Serratia*	*S. marcescens*	Carbapenems or aminoglycosides in combination with 3rd/4th generation cephalosporins	Wide spectrum of resistance to numerous antibiotic classes; biofilm formation complicates treatment	[288,289]
**Acid-fast bacteria**					
*Mycobacteriaceae*	*Mycobacterium*	*M. tuberculosis* complex	Isoniazid + rifampicin + pyrazinamide + ethambutol (standard tuberculosis treatment)	Emerging resistant strains may require more personalized or more prolonged therapeutic schemes	[162,290]
		*M. chelonae*	Levofloxacin + rifampin	Must be considered in chronic cases of infections with uncommon symptomatology; potential resistance after a few weeks of treatment	[291,292,293]
**Fungi**					
*Aspergillaceae*	*Aspergillus*	*A. fumigatus*	Voriconazole or amphotericin	Resistance patterns vary depending on different regions; widespread azole use has led to increasing resistance in different settings	[162,294,295]
*Debaryomycetaceae*	*Candida*	*C. albicans*	Fluconazole, amphotericin, or an echinocandin	In most cases, resistance rates are low, even though biofilm formation will increase resistance	[162,296,297]

MSSA = Methicillin-Sensitive *Staphylococcus aureus*, MRSA = Methicillin-Resistant *Staphylococcus aureus*.

The particular location of the infection possesses a significant constraint on the potential antibiotics to be administered. More specifically, the selected antibiotics must be characterized by adequate bone penetration. Regarding resistance to antibiotic agents, this is a well recognized and widespread problem in medicine; it is believed that about 10 million deaths will be attributable to antibiotic resistance by 2050 unless the extensive and incorrect use of such substances subsides [298]. This is exacerbated by the fact that those mostly affected by this phenomenon are immunocompromised patients, who are often in contact with the nosocomial setting, as well as the healthcare professionals who operate it [298,299]; the immunocompromised status is also a risk factor for spondylodiscitis, as previously mentioned.

Antibiotic resistance develops through a number of mechanisms, comprising transformation, transduction, and conjugation, which may induce the enzymatic inactivation of the pharmacological substance, decrease its penetration, activate efflux pumps, or lead to new protein synthesis; the molecular basis of these mechanisms has been analyzed by numerous researchers [298,299]. The bacteria that are mostly associated with antibiotic resistance are *Enterococcus* spp., *S. aureus*, *K. pneumoniae*, *A. baumannii*, *P. aeruginosa*, *Enterobacter* spp., *E. coli*, and *Salmonella* spp. [298,300]. In addition, the multi-drug resistant (MDR) strains mainly encountered in the context of the nosocomial setting are *S. aureus*, in the form of methicillin-resistant *S. aureus* (MRSA), *A. baumannii*, *P. aeruginosa*, and *K. pneumoniae* [299]. Based on the majority of the available evidence, the optimal antibiotic duration is 6 weeks, but can be extended for up to three months in cases of high-risk patients or complications [52,301]. In cases of fungal infections, the standard treatment duration is even longer [302,303]. However, antibiotics may be discontinued if the clinical improvement is noticeably positive and if the serum markers, most notably CRP, have normalized [304,305]. As of yet, there have been no notable controlled studies offering conclusive results as to the optimal treatment duration [225].

Seeing as *S. aureus* is so prevalent in the pathogenesis of infectious spondylodiscitis, the implications regarding therapeutic efficacy are worrisome. Currently, MRSA strains account from 10% to 30% [306] or even 45.50% of cases, are associated with a higher degree of dangerous complications, and necessitate reoperation six months and twelve months post-infection more frequently when compared to methicillin-sensitive *S. aureus* (MSSA) [307]. It should also be noted that pyogenic spondylodiscitis due to *S. aureus* is associated with high rates of treatment failure [308,309]. Regarding the other members of *Staphylococcus* spp., according to Fröschen et al. [43], 42.8% of all coagulase-negative staphylococci, which were involved in the pathogenesis of spondylodiscitis, were found to be oxacillin-resistant, though thankfully no vancomycin-resistant strains were detected among them. Besides MRSA, glycopeptide-resistant enterococci also pose a big therapeutic challenge [306]. MDR strains of Gram-negative bacteria should also not be discounted, as they can be occasionally encountered [306].

### 5.2. Surgical Treatment

Surgical intervention is reserved for cases where there are pronounced neurological deficits, spinal instability, or abscess formation, or when conservative treatment has proved ineffective [310]. The typical indications for surgical intervention are pronounced destruction with segmental kyphosis exceeding 15°, a vertebral body collapse of over 50%, or a translation of over 5 mm [3].

Starting from the SINS (Spinal Instability Neoplastic Score) score, a group of researchers has developed the SISS (Spinal Instability Spondylodiscitis Score) score, which showed high reliability and validity in detecting instability and evaluating treatment choices [311].

The presence of epidural empyema, especially if it is the cause of neurological deficits, is also considered an indication for surgical intervention, as per the guidelines [312]. These neurological deficits can have a wide range of manifestations, such as progressive weakness, sensory loss, or cauda equina syndrome, which are also indicators for a surgical approach [313,314].

The preference for surgery over conservative treatment is higher among surgeons the more pronounced the neurological deficits are [3]. Sepsis may be considered an indication for surgery in some cases, but, despite the treatment scheme elected, its presence is associated with increased mortality [125,315]. At any rate, the surgical treatment of spondylodiscitis, consisting of a staged surgical approach and a short period of intravenous antibiotics of two to three weeks, followed by three months of oral antibiotics, seems to be adequate when dealing with infections caused by multi-resistant bacteria in the majority of cases [306].

Regarding the operational approach itself, both open and minimally invasive techniques have been developed. The open approach is usually preferred in patients with abscess formation and in the presence of a motor deficit [316]. These approaches are also more likely to result in higher rates of radiologic bone union and more spinal stability compared to the minimally invasive techniques [310,317]; furthermore, the open techniques offer adequate surgical debridement [316]. Even despite these advantages, the existence of some drawbacks such as increased surgical trauma, greater blood loss, longer operative times, increased pain postoperatively, together with higher perioperative morbidity, especially in elderly and high-risk patients, make the minimally invasive approaches a better option in selected cases [316,317].

Minimally invasive techniques, on the other hand, are associated with reduced blood loss, shorter operative times, lower overall post-surgical pain and pain at discharge, and faster recovery [310,316,317]. In the absence of instability, patients in poor physical condition, with severe comorbidities, and those with bone destruction generally benefit the most from such approaches [318]. Moreover, these techniques can also be used in refractory spondylodiscitis [319]. However, minimally invasive techniques may result in lower rates of radiological bone fusion and provide less spinal stability [310,317]. Both approaches result in comparable clinical outcomes in terms of infection resolution and neurological recovery, but for advanced disease cases the open approach remains the preferred option by the surgeons [316].

### 5.3. Diagnostic Flowchart

**Figure 5:**
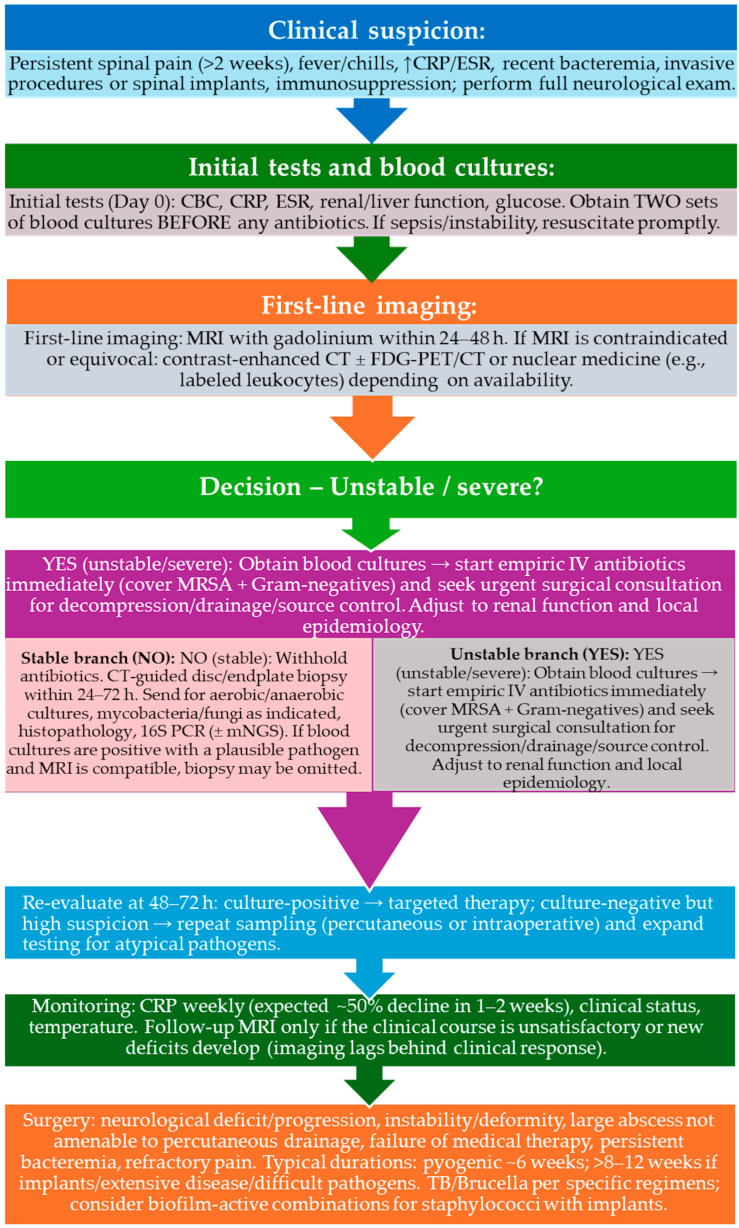
Diagnostic flowchart of adult infectious spondylodiscitis.

A schematic overview of the adult infectious spondylodiscitis diagnostic workup is presented in Figure 5.

## 6. Discussion

Infectious spondylodiscitis is a potentially serious and even life-threatening pathology, depending on the period between infection and diagnosis, on the identification of the pathogen, on the patient’s comorbidities, and on the treatment scheme selected. Its incidence has been on the rise lately, especially in developed healthcare systems, owing most probably to the ageing population, and also to the increased availability of accurate diagnostic methods. Lately, in the aftermath of the COVID-19 pandemic, there has been an increase in spinal infections reported in recovering patients alongside other complications [144,320,321,322].

Virtually every type of pathogen, with the exception of viruses, can be the cause of infectious spondylodiscitis, but the most commonly encountered types are the pyogenic form, caused by bacteria, and the tuberculous form, caused specifically by *Mycobacterium* spp. [71,99]. The pyogenic type of infectious spondylodiscitis may be caused by different bacterial pathogens, although the most commonly identified ones are *Staphylococcus* spp. [43,52]. The tuberculous type is most commonly associated with *Mycobacterium tuberculosis* complex [71]. The most common way of infection is hematogenous dissemination, usually by arterial spread [71].

The diagnosis is usually based on a combination of clinical symptoms, paraclinical investigations, and imaging findings [99]. From the clinical aspect, back pain and fever are the most common patient presentations [9,60]. Both discussed forms have very similar symptomatology, even though in the tuberculous form, the patients frequently report a longer symptom duration; another differentiating aspect is that the pyogenic form frequently affects two vertebrae, while the tuberculous form tends to affect three [81]. While a set of non-specific biochemical markers, such as CRP and ESR, coupled with a suggestive symptomatology, can point towards the diagnosis, based on a review of research evidence, specific markers may be of use in diagnosing TB spondylodiscitis in particular [323].

When it comes to the use of imaging in the diagnosis of infectious diseases, there are difficulties in the initial diagnosis, depending on the stage of the infection [324,325,326]; differential diagnoses may also present a challenge [327,328,329]. Specifically for infectious spondylodiscitis, there are different methods available, each one with its advantages and disadvantages. Bone scintigraphy, plain X-ray, and CT can all provide suggestive images at different stages of the disease, but MRI is considered to be the gold standard of imaging [99]; it is also useful as it may provide clues as to the nature of the diseases, i.e., tuberculous or non-tuberculous [81]. Care must be taken in the imaging of associated conditions, such as spondylitis without discitis, seen in initial infection stages or in immunocompromised elderly patients [330], and facet joint infections in the zygapophyseal joints [33,124,331].

Microbiological methods and MRI findings are vital components for the proper diagnosis of pyogenic spondylodiscitis. Research findings suggest that molecular methods, such as the clinical application of 16S rRNA PCR and sequencing, may be useful as adjunctive diagnostic tools for pyogenic spondylodiscitis. The rapid turnaround time of 16S rRNA PCR and sequencing submission and results can potentially decrease the time to diagnosis and improve the therapeutic management and outcome of these infections [51]. A newer method, mNGS, is also very rapid and accurate, but it is very high cost, which prohibits its regular use [132]. Nevertheless, it is invariably true that microbiological diagnostic methods have generally reduced sensitivity, specificity, and accuracy the longer the antimicrobial treatment is [128]. Irrespective of the positive identification of the pathogen, sometimes empirical treatment will have to start, as in infections, the earlier the treatment initiation, the better the outcome [332].

Blood cultures play a key role, yielding positive results in half of the pyogenic cases. When they turn up negative, tissue directly from the site of infection may be required. Open biopsies provide a higher diagnostic yield than percutaneous CT-guided biopsies, even though they are more invasive. The timing of spinal biopsy and the optimal method in each case [234,333] add another layer of complexity related to the diagnosis of infectious spondylodiscitis. Empirical therapy is required in one-third of cases [9], even after all available diagnostic means have been exhausted.

Given the vast difference in clinical manifestations and aetiologies, along with the applied treatment schemes, it is to be expected that patient outcomes may differ significantly, but mortality rates remain invariably high [17]. The treatment can be conservative or surgical, the latter being preferred in the case of neurological deficits or instability [11]. On the other hand, conservative treatment is the mainstay in most situations. Either way, there is a dearth of consensus on the optimal treatment, not helped by the few relevant clinical trials and the absence of definitive guidelines [70,162]. More recent data seem to suggest that early surgical interventions may offer improved patient outcomes and reduced mortality [334,335].

The Infectious Diseases Society of America recommends a total duration of 6 weeks of parenteral or highly bioavailable oral antimicrobial therapy for most patients with bacterial native vertebral osteomyelitis (spondylodiscitis). This recommendation is based on a landmark randomized controlled trial demonstrating that 6 weeks of antibiotic treatment is non-inferior to 12 weeks, with similar cure rates of approximately 91% in both groups. The exception is spondylodiscitis due to Brucella species, which requires 3 months of antimicrobial therapy [312]. The standard antibiotic duration is also supported by high-quality evidence from a multicenter randomized controlled trial of 351 patients that demonstrated non-inferiority of 6-week versus 12-week treatment (cure rates 90.9% in both groups). This shorter duration reduces antibiotic-related adverse events, healthcare costs, and antibiotic resistance without compromising outcomes [336]. However, certain organisms may require longer treatment: *S. aureus* infections may benefit from at least 8 weeks, and MRSA infections treated with daptomycin should receive 8 weeks of therapy [337].

Regarding surgical intervention, the Infectious Diseases Society of America notes that the majority of patients can be cured with antimicrobial therapy alone, though some may require surgical debridement and/or spinal stabilization during or after treatment. Indications for surgery include the development of neurologic deficits or symptoms of spinal cord compression, and evidence of progression or recurrence despite proper antimicrobial therapy. The guidelines emphasize that except in septic patients or those with neurologic compromise, empiric antimicrobial therapy should be withheld when possible until a microbiologic diagnosis is confirmed through image-guided or intraoperative aspiration or biopsy [312].

Regarding surgical treatment options, while there exist guidelines regarding the parameters that have to be met for a surgical intervention to be considered, different medical professionals may choose to intervene sooner than recommended, based on their medical judgement and patient requests [3]. Regardless of the time of surgical intervention, in order to replace a part of the destroyed vertebra, implants may be used [314]. In such cases, the use of 3D printing, with its increasing application in orthopedics, should be considered [338,339,340,341,342]. Relevant developments allow, apart from the reliable and effective replacement of bone and cartilage parts [343], the incorporation of antimicrobial agents, both antibiotics [344] and phytochemicals with antimicrobial potential [345,346,347,348,349,350,351,352]; these have the potential to improve the probabilities for a successful surgical outcome, target the infection in situ, and improve post-operative rehabilitation.

The choice between surgical and conservative treatment remains controversial and depends on specific clinical indications. Recent evidence presents conflicting findings: Arguments favouring early surgery: A 2023 meta-analysis of 10,954 patients found that early surgical intervention was associated with a 39% reduction in mortality (8% vs. 13%), 40% reduction in relapse/failure rates (15% vs. 21%), and 7.75 days shorter hospital stay compared to conservative management [353]. A 2024 propensity-matched study similarly showed significantly lower mortality with surgery (4.2% vs. 24.2%) [354].

There are arguments supporting a conservative management. A 2025 meta-analysis found no significant difference in mortality between surgical and conservative treatment, with outcomes heavily influenced by patient selection and comorbidities [355]. A 2024 retrospective study of 112 patients with neurologically intact pyogenic spondylitis found no significant differences in hospital stay, antibiotic duration, complications, or recurrence between groups, though surgery provided better correction of kyphotic deformity [356].

Patients with neurological deficits or spinal cord compression represent the clearest indication for surgery, with evidence showing that surgical patients achieve faster pain control, earlier mobilization, and shorter hospital stays despite starting from worse clinical conditions [357]. The presence of an epidural abscess on MRI is a crucial predictor requiring early surgical intervention, with one study showing significantly higher incidence of an epidural abscess in surgical versus non-surgical groups (*p* = 0.001) [358]. Younger, less frail patients appear to derive greater benefit from surgery. A study comparing operative versus conservative management found that surgical patients were significantly younger (62.9 vs. 70.7 years) and less frail (modified Frailty Index 1.09 vs. 1.85), with surgical intervention associated with lower 30-day mortality (2.3% vs. 17.8%, *p* = 0.016). However, patients with a modified Frailty Index >3 had significantly higher 30-day mortality (30.4% vs. 7.5%) regardless of intervention, suggesting extreme frailty may limit surgical benefit [359]. Patients with spinal instability, vertebral collapse, or severe intractable pain benefit from surgery, with evidence showing better correction of kyphotic deformity and faster pain relief compared to conservative management [357]. Cervical spondylodiscitis, vertebral collapse, and epidural collections are associated with a higher risk of neurological deficits, warranting closer consideration of surgical intervention [360]. Neurologically intact patients without instability can be successfully managed conservatively, with similar recurrence rates and potentially lower complication rates compared to surgery [337]. A 2024 study of 112 neurologically intact patients found no significant differences in hospital stay, antibiotic duration, complications, or recurrence between surgical and conservative groups [356]. Frail patients may benefit from stabilization without aggressive debridement when surgery is necessary, as extensive procedures carry higher perioperative risk. The decision should be individualized based on frailty status, comorbidities, and response to initial antibiotic therapy [314].

Modern management of spondylodiscitis includes a multidisciplinary team, although this is not always possible in different settings [361]. A team formed by an infectious disease specialist, a spine surgeon, a radiologist, and a microbiologist is needed for the best approach regarding adequate diagnosis and treatment [362].

This work provides a pragmatic, imaging-led diagnostic pathway for adult infectious spondylodiscitis, but several limitations should be acknowledged. First, the recommendations are synthesized from heterogeneous observational studies, expert guidance, and institutional practice rather than randomized trials; hence, some steps remain consensus-based and context-dependent. Second, diagnostic accuracy varies by timing and availability of tests: early MRI can be equivocal, PET/CT and nuclear medicine studies are not universally accessible, and inter-observer variability may affect the interpretation of Modic 1-like changes versus infection. Third, microbiological yield is influenced by prior antibiotic exposure, sampling technique, and laboratory protocols (aerobic/anaerobic culture conditions, incubation time, mycobacterial and fungal work-up); even with meticulous technique, culture-negative disease is common, and molecular assays (16S rRNA PCR and mNGS) introduce their own constraints (contamination risk, cost, turnaround time, and a lack of phenotypic susceptibility results). Fourth, the algorithm privileges an MRI-first approach and blood cultures before antibiotics; in unstable patients this sequence may not be feasible, and urgent source control justifiably precedes complete diagnostics. Fifth, proposed empiric regimens, durations, and the use of biofilm-active combinations should be adapted to local epidemiology and antimicrobial resistance patterns; generalizability to settings with high TB/*Brucella* prevalence or limited laboratory/imaging capacity is restricted. Sixth, special populations (immunocompromised hosts, chronic renal failure, advanced deformity, or extensive instrumentation) may require departures from the pathway; pediatric spondylodiscitis is outside the scope. Seventh, the pathway has not yet undergone prospective, multicenter validation; its clinical utility, safety, and cost-effectiveness should be tested against usual care and audited with predefined quality indicators (time to diagnosis, pathogen yield, unplanned surgery, and relapse). Finally, imaging and laboratory follow-up recommendations (e.g., CRP decline and selective use of repeat MRI) are based on observational kinetics and may not uniformly predict outcomes; clinicians should integrate patient trajectory, neurological status, and source-control adequacy when making decisions.

## 7. Conclusions

Infectious spondylodiscitis of bacterial origin is characterized by rising incidence with a difficult diagnosis due to its non-specific presentation and frequent lack of initial definitive findings. MRI remains essential for imaging assessment, but accurate diagnosis requires the integration of clinical, laboratory, and microbiological data. Furthermore, microbiological confirmation is essential, despite the limitations of current molecular techniques which may complicate the etiological diagnosis.

Treatment should be individualized according to the pathogen profile, severity of the infection, the presence of neurological deficits or spinal deformities, and other host-related factors. The surgical intervention will not obviate the need for a prolonged antibiotic administration but may reduce the length of administration and offer better clearance of the infected area and correction of associated deficits. Future improvements in the field may lead to a quicker diagnosis, improved microbiological yield, and may provide better treatment and follow-up strategies, as well as improved patient outcomes.

## Data Availability

No new data were created or analyzed in this study. Data sharing is not applicable to this article.

## Abbreviations

The following abbreviations are used in this manuscript:

16S rRNA	16S ribosomal RNA
AFB	Acid-fast bacilli
ALP	Alkaline phosphatase
CBC	Complete blood count
COVID-19	Coronavirus disease 2019
CRP	C-reactive protein
CT	Computed tomography
DNA	Deoxyribonucleic acid
ESR	Erythrocyte sedimentation rate
FDG-18	Fluorine-18-fluorodeoxyglucose
MDR	Multiple drug resistance
mNGS	Clinical metagenomic next-generation sequencing
MRI	Magnetic resonance imaging
MRSA	Methicillin-resistant Staphylococcus aureus
MSSA	Methicillin-susceptible staphylococcus aureus
PCR	Polymerase chain reaction
PET	Positron emission tomography
SAPHO	Synovitis, acne, pustulosis, hyperostosis, and osteitis
SE	Spin-echo
SPECT	Photon emission computed tomography
SINS	Spinal instability neoplastic score
SISS	Spinal instability spondylodiscitis score
